# Biomolecular Characterization of *Trichomonas vaginalis* from Spain: Evaluating Genetic Correlation with Drug Resistance and Endobionts

**DOI:** 10.3390/biology14060618

**Published:** 2025-05-27

**Authors:** Alexandra Ibáñez-Escribano, Juan José Nogal-Ruiz, José Antonio Escario, Francisco Ponce-Gordo

**Affiliations:** Department of Microbiology and Parasitology, Faculty of Pharmacy, Universidad Complutense de Madrid, 28040 Madrid, Spain; jjnogalr@ucm.es (J.J.N.-R.); escario@ucm.es (J.A.E.); pponce@ucm.es (F.P.-G.)

**Keywords:** *Trichomonas vaginalis*, genotypes, TVV, *Mycoplasma*, resistance, microsatellites, single-copy gene, SNP

## Abstract

*Trichomonas vaginalis* causes one of the most prevalent sexually transmitted infections (STIs). Despite extensive biomolecular research on this protozoan, no efficient molecular tool currently exists for the intraspecific classification of *T. vaginalis* isolates. In recent years, an incipient tendency has been observed to classify this parasite into two genotypes that correlate in a high percentage with phenotypic characteristics (such as metronidazole resistance and viral endobionts). However, this grouping method has not yet been consolidated by the scientific community. In this sense, *Mycoplasma hominis* has also been identified as an endobiont of *T. vaginalis*, but no previous studies on its presence and possible correlation with the two *T. vaginalis* populations detected have been executed. In the present study, several new *T. vaginalis* isolates of Spanish origin have been characterized using two molecular markers (microsatellites and single-copy genes), the presence of *T. vaginalis* virus (TVV) and *Mycoplasma*, and the resistance to reference drugs. On the basis of our molecular results, the isolates were classified into two groups, as proposed by other researchers. In relation to the biological determinations, the two isolates harboring *Mycoplasma* were sensible to the reference drugs and were included in the same genotypic group. Although the presence of TVV was more notable in one group than in the other, both groups had TTV+ and TVV− isolates. These findings indicate that genetic grouping does not strongly correlate with susceptibility to reference drugs or endobiont presence, suggesting the need for further research into alternative classification models.

## Simple Summary

*Trichomonas vaginalis* is the causative agent of over 40% of curable sexually transmitted infections worldwide. Despite extensive research, the impact of *T. vaginalis* genetic variability and its endobionts (viruses and *Mycoplasma* sp.) on clinical presentations and drug resistance remains unclear. To address this gap, we conducted a study combining phenotypic analysis with molecular fingerprinting. The phenotypic findings, and the molecular characterization yielded results consistent with previous studies using similar biomarkers. The isolates were classified into two types, in line with the findings of other researchers. However, no biomolecular tool currently exists that can classify *T. vaginalis* with 100% accuracy in terms of drug sensitivity, pathogenicity, clinical manifestations, or the presence of endobionts.

## 1. Introduction

*Trichomonas vaginalis* is a leading cause of non-viral sexually transmitted infections (STIs), with an estimated annual incidence exceeding 156 million cases [1]. The causative agent of this STI is a protozoan with a microaerophilic (or aerotolerant) metabolism, responsible for trichomoniasis, a disease with a broad spectrum of clinical manifestations. Infected women usually range from asymptomatic cases (85%) to mild symptomatic ones including vaginal discharge, vulvar itching, vaginitis, and dysuria [2]. In more severe instances, the infection can lead to invasion of the genitourinary ducts, including Bartholin’s glands, fallopian tubes, and Skene’s glands, as well as parenchymal colonization and transient infertility [3]. *Trichomonas vaginalis* infection has been linked to significant adverse reproductive health outcomes. In pregnant women, trichomoniasis is associated with an elevated risk of preterm delivery, premature rupture of membranes, and low birth weight [4]. Furthermore, *T. vaginalis* infection has been implicated in increasing the risk of cervical cancer [5]. Furthermore, the implications of this STI increasing the risk of transmission and acquisition of other STIs (papillomavirus, HIV, among others) have been extensively demonstrated in recent years [2].

Clinical isolates may consist of several heterogeneous subpopulations, influenced by the physiological conditions of the patient or the post-isolation maintenance of the parasite [6]. Additionally, possible genetic exchange in the parasite has been suggested [7]. These peculiarities in reproduction and population structure may explain the genetic diversity and the phenotypic heterogeneity of *T. vaginalis* isolates. On the other hand, the draft *T. vaginalis* genome is a ~160 Mb assembly with a core set of ~60,000 protein-coding genes with 65% of repeated and transposable elements, as well as a high number of homologous proteins with bacteria, viruses, and parasites [8]. The complex structure of this genome suggests an abundant presence of microsatellites (MSs)—short tandemly repeated DNA sequences located at specific loci—and a comparatively lower representation of single-copy genes (SCGs), which are not duplicated across the genome. These characteristics make both elements particularly valuable as molecular biomarkers [9].

The high genetic variability and large genome size of the parasite have hindered efforts to establish a consistent link between genotype and phenotype, such as pathogenicity and drug resistance. Moreover, it is likely that the variable pathobiology of this STI is influenced by a range of extrinsic and intrinsic factors, including both biological and genetic aspects of the parasite and the host.

Besides the intrinsic genetic variability, *T. vaginalis* can harbor endobionts such as *T. vaginalis* virus (TVV), the first dsRNA virus described in protists [10]; or mycoplasmas (*Mycoplasma hominis* and *Candidatus* Mycoplasma girerdii) [11], designated as the first symbiosis between two human pathogens living in the same niche [12]. The role of these viral and bacterial endobionts in the pathobiology of *T. vaginalis* remains unclear, with conflicting results in the literature [13,14,15]. However, experts agree that these endobionts may have a significant impact on the virulence of trichomoniasis [16] and consequently on the diagnosis and treatment of the disease [11].

Previous researchers have attempted to use molecular tools to characterize *T. vaginalis* isolates in terms of clinical manifestations [17] or the presence of TVV [18]; however, other groups have not been able to correlate the same findings [19]. Furthermore, the lack of results from other research groups concerning molecular and biological characterizations hinders the discovery of a molecular fingerprint that permits the association of a molecular probe with pathogenicity or any other type of biological feature, including drug resistance, presence of endobionts, and so forth. Conrad et al. (2012) [7] have proposed to classify the isolates into two genotypes, namely 1 and 2, according to a panel of 21 MSs and six SCGs. Type 1 isolates were usually associated with the presence of TVV, while Type 2 isolates exhibited greater resistance to metronidazole. The main disadvantage of the panel proposed by Conrad et al. (2011, 2012) [7,9] is the extensive number of loci, which increases the cost associated with the molecular analysis.

In this context, the primary objective of this study is to genotype several *T. vaginalis* isolates using a limited set of molecular markers (three MSs and two SCGs) and to evaluate whether this genetic profile aligns with the two types proposed by other researchers. Additionally, phenotypic characterization of these isolates has been conducted, assessing their resistance to 5-nitroimidazoles and the presence of endobionts, including TVV and *Mycoplasma* sp. The subsequent discussion addresses the utility of the markers selected for the classification of isolates.

## 2. Materials and Methods

### 2.1. Trichomonas vaginalis Isolates

Ten *Trichomonas vaginalis* isolates were included in this study. Isolates JH31A4 and IR78 were obtained from the American Type Culture Collection, while S/H, 1807, 1232, 11, S019, S760, S852, and S351 were isolated from female patients attending health centers of the Health Care System of the Community of Madrid (Madrid, Spain) and cryopreserved in liquid nitrogen. The obtention of the samples and their determinations were ethically approved by the Research Ethics Committee of the Hospital Universitario Puerta de Hierro Majadahonda-Madrid (granted permission Acta no. 21.17), adhering to the Declaration of Helsinki. All the samples were cultured in TYM modified medium supplemented with 10% of heat-inactivated fetal bovine serum (FBS) and antibiotics (100 UI/mL penicillin, 100 μL/mL streptomycin, and 100 μL/mL gentamicin) and incubated at 37 °C and 5% CO_2_. Subcultures were made every 48–72 h.

### 2.2. DNA Extraction

For the molecular studies, total DNA was extracted from *T. vaginalis* mid- to late-logarithmic phase axenic cultures with the Speedtools DNA Extraction Kit (Biotools, Spain) following the manufacturer’s recommendations. The total DNA was used immediately or conserved at −20 °C until used.

### 2.3. Microsatellite Loci Genotyping

From the MS markers proposed by Conrad et al. (2011, 2012) for *T. vaginalis* typing, MS06, MS129, and MS184 were selected according to the following criteria: presence of five or more alleles with different lengths between the amplicons to be obtained. The SCGs selected were leishmanolysin-like metallopeptidase (GP63a) and a mismatch repair MutL. Homolog (PMS1), which, according to published results, had the highest number of single nucleotide polymorphisms (SNPs) among those used in *T. vaginalis* characterization [7,9].

The primers and conditions used for the PCR amplification of the MS markers were those described by Conrad et al. (2011) [9]. Briefly, “tail” primer 5′-GTCGTTTTACAACGTCGTG-3′ was either labeled with HEX or 6-FAM phosphonamidite conjugates or added to the 5′ end of forward primers. The list of tail-forward and reverse primers used is indicated in Table 1. Amplifications were performed using the kit PurReTaq^TM^ Ready-To-Go^TM^ PCR beads (Merck KGaA, Darmstadt, Germany) in a total volume of 25 µL, containing 1 µM of labeled-tail primer, 50 nM of tail-forward primer, 1 µM of reverse primer, and 5 µL of template DNA. The PCR reactions were made on an Eppendorf Master Cycler Gradient (Eppendorf AG, Hamburg, Germany) programmed as indicated in Table 1. The resulting products were sent to the Sequencing Service of the Complutense University, where they were separated by capillary electrophoresis on an A3730xIR DNA Analyzer (Applied Biosystems) with GeneScan™ 500 LIZ™ Size Standard (Applied Biosystems) for size determination. The size of the MS amplicons was determined with the Peak Scanner v 1.0 software (Applied Biosystems).

### 2.4. Single-Copy Gene Loci Genotyping

Own-designed primers enabled partial amplification of the *GP63a* and *PMS1* genes (Table 2). PCR conditions were as described by Conrad et al. (2011) [9], with some modifications. Briefly, amplifications were performed using the kit PurReTaq^TM^ Ready-To-Go^TM^ PCR beads in a total volume of 25 µL, containing 2 µL of each forward and reverse primer and 5 µL of template DNA. The thermocycler (Eppendorf Master Cycler Gradient) was programmed as follows: initial denaturation, 95 °C for 3 min, followed by 30 cycles of denaturation at 95 °C for 45 s, primer annealing at 55 °C (GP63a) or 60 °C (PMS1) for 1 min, extension at 72 °C for 2 min, and a final extension at 72 °C for 7 min. PCR products were visualized in 1% agarose gels stained with Pronasafe (Condalab, Torrejón de Ardoz, Spain), purified with QIAquick^®^ PCR Purification Kit (Qiagen, Hilden, Germany), and sequenced in both senses in an 3730xl DNA Analyzer (Applied Biosystems). The chromatograms were edited and assembled in ChromasPro ver. 2.1.8 (Technelysium Pty Ltd., South Brisbane, Australia) to obtain the complete amplified fragment for each isolate.

### 2.5. Phylogenetic Analysis

Sequence alignment and comparisons were carried out in MEGA-X ver. 10.0.5 [20]. For this purpose, we have included the GP63a and PMS1 sequences of the six isolates that have been characterized for both SCG by Conrad et al. (2011, 2012) (accession numbers HM365121-26 for GP63a; HM365174-78, JN380562, JN380585, JN380593, JN380596, JN380599, and DQ321767 for PMS1) [7,9]; the 5′ and 3′ ends of the alignments were trimmed to adjust the length of all the sequences. Phylogenetic trees were inferred by the Neighbor-Joining method; the Tamura 3-parameter model was selected as the best model of nucleotide evolution based on the Akaike information criterion and the Bayesian information criterion calculated in MEGA-X. The data were bootstrap resampled 1000 times to estimate the relative branch support. All GP63a and PMS1 sequences have been deposited in the GenBank database under accession numbers PQ540993-PQ541012.

### 2.6. Drug Resistance to 5-Nitroimidazoles

Drug resistance to metronidazole and tinidazole was determined following the method described by Bolumburu et al. (2020) with slight modifications [21]. Briefly, trichomonads were seeded at a final concentration of 10^5^ trophozoites/well in microtiter plates. The viability of the trophozoites was determined after 48 h of incubation with different drug concentrations (512 to 2 µg/mL) at 37 °C and 5% CO_2_, using the resazurin fluorimetric method previously standardized in our lab [22]. The plates were incubated with redox dye (3 mM stock solution in PBS 1X) for 1 h at 37 °C and 5% CO_2_. Then, the plates were read in a fluorometer (Infinite 200, TECAN) at λex 535 nm and λem 590 nm. *Trichomonas vaginalis* isolates were classified as metronidazole (MTZ)- and tinidazole (TDZ)-resistant according to Kirkcaldy et al.’s (2012) criteria, defined when aerobic minimal lethal concentration (MLC) ≥ 50 µg/mL [21,23].

### 2.7. Determination of Trichomonas vaginalis Virus

Indirect immunofluorescence (IFI) confirmed the presence of TVV, using the monoclonal antibody (MoAb) C20A3 [24] kindly provided by Professor J.F. Alderete. The ten isolates were fixed in 2% formaldehyde solution and posteriorly treated with acetone for 10 min at 4 °C using IFI slides (Biomérieux, France). Then, trophozoites were first incubated with the MoAb C20A3 (1:100) for 1 h at 37 °C and then with the secondary antimouse IgG-FITC (1:200). Samples were stained with Evans blue and visualized in a fluorescence microscope (Olympus BH2) [21].

### 2.8. Determination of Mycoplasma hominis

Amplification of a 16S rRNA fragment of *Mycoplasma* spp. from the total DNA extracted from the samples, using the Mycoplasma Detection Kit (Southern Biotech, Birmingham, AL, USA), enabled detection of *M. hominis*, following the manufacturer’s protocol and including the positive and internal negative controls provided in the kit. Amplification products of 503 bp corresponding to positive samples, and of 270 bp corresponding to internal controls were visualized in 1.5% agarose gels stained with Pronasafe (Condalab, Spain) under UV illumination.

## 3. Results

### 3.1. Microsatellites

The MS06 marker showed a standard length of 395 bp in most isolates, with slight variations in IR78 (412 bp) and S852 (407 bp). The amplicons analyzed for MS129 showed a constant length of 192 bp, except for S019 and S852, which displayed smaller amplicons differing by seven and eight nucleotides, respectively. For MS184, with a constant length of 250 bp, two exceptions were observed: isolates 1232 and S019, with 241 bp and 253 bp, respectively (Table 3).

The MS amplicons allowed the identification of two distinct groups: group MS-A composed of JH31A4, S/H, 1807, 11, S760, and S351, which showed a uniform nucleotide length across all three MSs. Group MS-B, consisting of 1232, IR78, S019, and S852, presents one or two MSs with different sizes, as shown in Table 2.

### 3.2. Single-Copy Genes

#### 3.2.1. GP63a

**Figure 1:**
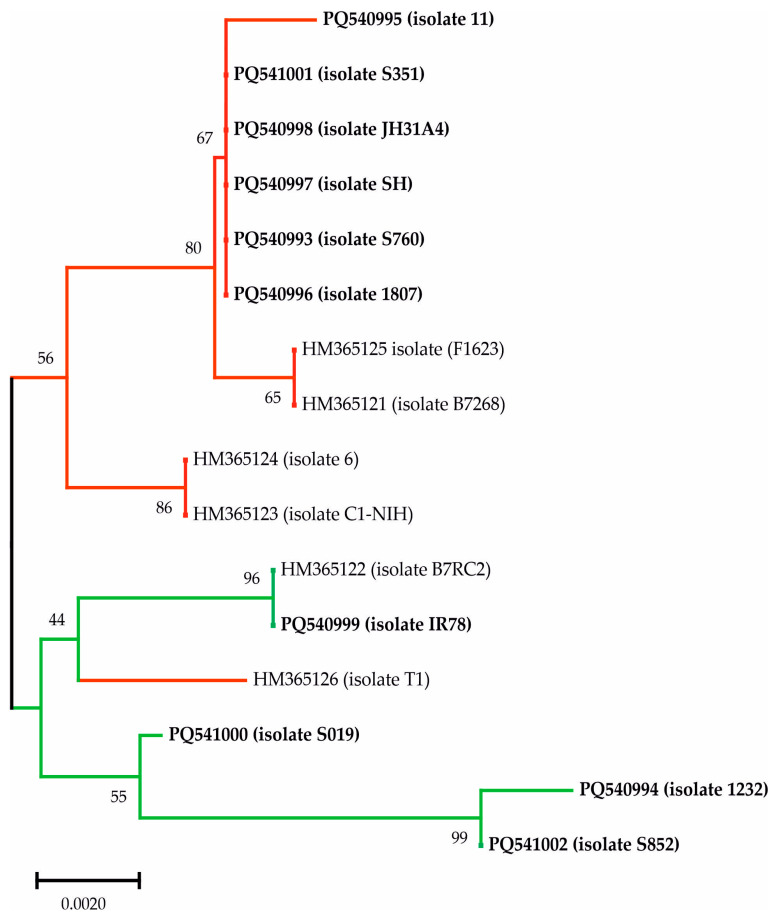
Phylogenetic relationships of the GP63a sequences of *Trichomonas vaginalis* by Neighbor-Joining method. The number at the nodes represents the bootstrap support as computed from 1000 replicates. The tree is drawn to scale, with branch lengths measured in the number of base substitutions per site. Isolates grouped as GP63a-A or Type 1 are shown in red and GP63a-B or Type 2 in green. The sequences obtained in the present study are in bold.

The GP63a alignment exhibited a significant variation with 14 potential SNPs, although two of them were found only once. Isolates JH31A4, S/H, 11, 1807, S351, and S760 were identical or almost identical and were grouped as GP63a-A, while the remaining isolates with a greater number of differences were considered as GP63a-B (Table 2; Supplementary File S1). These groupings were maintained for the sequences obtained in this study after construction of the phylogenetic trees, which included the isolates published by Conrad et al. (2011, 2012) [7,9]. The isolates grouped as Type 1 by Conrad (2012) were mostly grouped in GP63a-A, and most of the Type 2 sequences were clustered in GP63a-B. Only the HM365126 (isolate T1) sequence, considered as Type 1 [7], was included in the branches with the GP63a-B isolates, and the HM365125 (isolate F1623) Type 2 was included in the GP63a-A branches (Figure 1). Table 4 exhibits the data on isolates published in other studies [7,9] and used in phylogenetic comparisons with our clinical isolates. This table shows the population type and biological characteristics (resistance to metronidazole and presence of TVV) described by [7,9], and in which cluster they were grouped in our results. These data reflect no exact correlation between the type described by Conrad and the presence/absence of TVV or the group (A or B) obtained according to our studies with the two SCGs.

#### 3.2.2. PMS1

**Figure 2:**
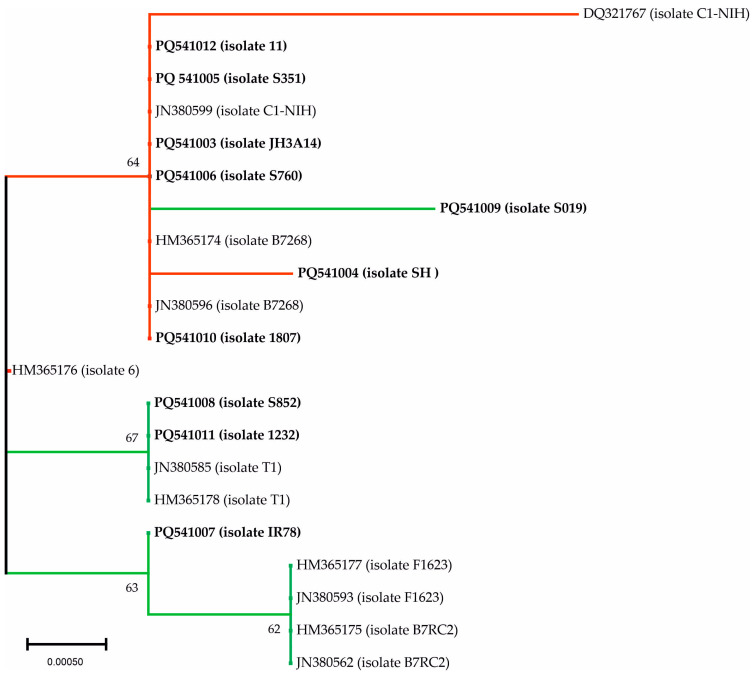
Phylogenetic relationships of the PMS1 sequences of *Trichomonas vaginalis* by Neighbor-Joining method. The number at the nodes represents the bootstrap support as computed from 1000 replicates. The tree is drawn to scale, with branch lengths measured in the number of base substitutions per site. Isolates grouped as PMS1-A or Type 1 are shown in red and PMS1-B or Type 2 in green. The sequences obtained in the present study are in bold.

The alignment of this gene revealed 10 positions with SNPs, although six of them were found only once. In terms of homology between the isolates sequenced in this study, six were identical (one isolate had only one base difference) and were grouped as PMS1-A, while the other sequences with two or more differences were classified as PMS1-B (Table 2; Supplementary File S2). In terms of phylogenetic studies, two populations were clearly identified (Figure 2). The isolates grouped as PMS1-A and PMS1-B were located on different branches, except for S019 (PMS1-B), which was located with the PMS1-A sequences. Sequences JN380585 and HM365178 (isolate T1), classified as Type 1 [7], were grouped with Type 2 and PMS1-B isolates (Table 3; Figure 2).

### 3.3. Biological Characterization

#### 3.3.1. Drug Susceptibility

Nine samples showed susceptibility to both 5-nitroimidazole drugs, with an MLC of ≤25 µg/mL. Only IR78 demonstrated a high level of resistance to metronidazole, with an MLC_MTZ_ > 400 µg/mL, while exhibiting a low-level resistance profile to tinidazole (MLC_TDZ_ = 50–100 µg/mL) (Table 2).

#### 3.3.2. TVV Presence

**Figure 3:**
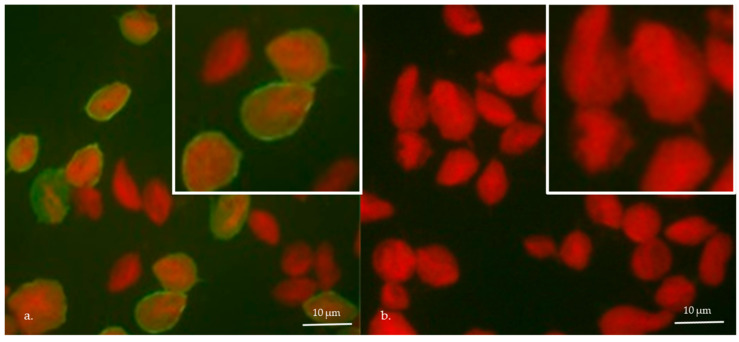
Determination of the presence of TVV endobiont in *T. vaginalis* isolates by indirect immunofluorescence using the anti-P270 monoclonal antibody C20A3: (**a**) Representative isolate positive for TVV, exhibiting green fluorescence on the cell surface; (**b**) Representative isolate negative for TVV, displaying red fluorescence from Evans blue staining.

Sixty percent of *T. vaginalis* isolates (JH31A4, S/H, 1807, 11, S019, and S760) expressed P270 on the cell surface, and were therefore classified as TVV-positive. In contrast, 1232, IR78, S351, and S852 were classified as TVV-negative (Table 2; Figure 3).

#### 3.3.3. *Mycoplasma* Presence

**Figure 4:**
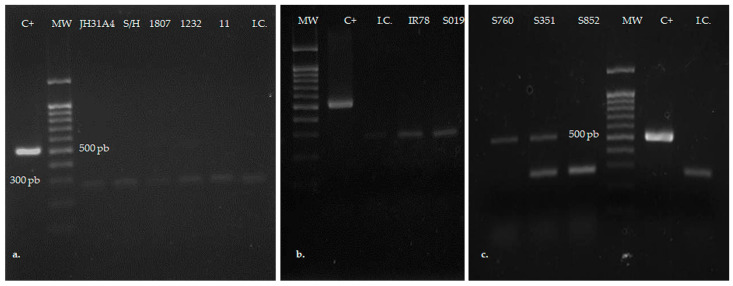
Determination of the presence of *Mycoplasma* sp. endobiont in *T. vaginalis* isolates by the identification of bacterial DNA: (**a**,**b**) Negative isolates, showing a 270 pb band corresponding to the internal control (I.C.); (**c**) The two positive isolates to *Mycoplasma* (S760 and S351), showing a positive band at 500 pb.

The molecular determination of mycoplasmas harbored by *T. vaginalis* isolates revealed that two out of 10 of the isolates studied were found to carry *Mycoplasma* (Figure 4). The presence of both TVV and *Mycoplasma* was only detected in isolate S760.

## 4. Discussion

Numerous attempts to establish an accepted fingerprint marker for genotyping *T. vaginalis* have been conducted without success. Other protozoans are classified into different genotypes; nevertheless, no unified classification has been established for *T. vaginalis*. The extent of heterogeneity observed in clinical isolates has been widely demonstrated in both biological and genetic studies [17,25,26]. Therefore, and in an attempt to search the best genomic fingerprints to characterize subtypes of this heterogenous parasite, different molecular tools have been tested for this purpose, with controversial results [17,18,19,27]. In recent years, different research groups have initiated an emerging trend of grouping *T. vaginalis* into two separate genotypes using different molecular markers, including RFLP, RAPD, MS, SNP, and MLST [7,18,28,29,30]. Some of them have also associated these genotypes with specific phenotypic traits: Type 1 isolates show an increased sensitivity to metronidazole and are more likely to harbor TVV in contrast with Type 2 isolates [7,18,30]. It is remarkable to indicate that the TVV criteria are not always accomplished. In other words, some isolates genotyped as Type 1 and 2 in these studies did not always achieve both biological features. The study conducted by Snipes et al. (2000) using the RAPD method classified several isolates in two clear lineages, showing a statistical correlation between not harboring TVV and those more likely to be resistant to MTZ [18]. However, one *T. vaginalis* TVV+ isolate was grouped with Type 2 isolates. The same applies to the classification by Conrad et al. (2012), with more than one isolate incorrectly associated for both groups, i.e., isolates considered as Type 1 but without TVV (this occurs in 20 isolates out of the 159 analyzed) and Type 2 isolates which are TVV+ (i.e., NYCE33 and SS-11 isolates) [7]. It is noteworthy that TVV could be acquired and lost several times in an isolate [31], or after long-term cultivation [32], as reflects the fact that the reference isolate C-1:NIH (ATCC 30001) characterized by Vanacova et al. (1997) presented TVV, while the results obtained by Conrad et al. (2012) with the same isolate exhibited no TVV, suggesting the loss of the viral endobiont [7,31]. Regarding the association between genotype and resistance to 5-nitroimidazoles, these authors defend that Type 2 isolates are more likely to be less sensitive to the drug [7,18,30]. However, as occurs with TVV, being clustered as Type 2 does not necessarily imply a resistant isolate. For instance, B7RC2, which was grouped as Type 2, exhibited a metronidazole MLC = 2 µg/mL [7]. Nonetheless, Conrad et al. (2012) detected a highly significant difference between the mean of MLC exhibited by Type 1 isolates vs. Type 2 [7].

It is clear that, to date, all researchers who have studied this complex parasite have not identified a molecular marker that can be associated with any biological or clinical characteristic with 100% accuracy. Analyzing our results and those of other authors, a complex population structure is consistently observed, which may be a consequence of the potential heterogeneous mix of subpopulations. This heterogeneity seems to explain the observed variability and the differences reflected in the different studies, making it challenging to establish definitive characterization criteria.

Our results are a clear reflection of what has been explained above. Firstly, the classification into two clear groups (A and B) is in agreement with the aforementioned studies [7,18,28,29,30]. Secondly, this classification, based on the nucleotide length of the three MSs and the alignments of the two SCGs studied (GP63a and PMS1), is almost consistent for our isolates. Only S019, which was grouped as B by GP63a and MS studies, was placed on a separate branch with the isolates in group A; curiously, this isolate was TVV+. Nevertheless, these discrepancies have also been detected in the analysis of the molecular and biological features characterized by Conrad et al. (2011, 2012) and which have been used in this study for the comparison with our isolates [7,9]. In this sense, isolate T1, considered by these authors as Type 1, was grouped in the branches with our group B isolates. Moreover, isolate F1623 (Type 2) was included in the group A branches for GP63a but with group B isolates with PMS1.

Furthermore, we have included the presence of *Mycoplasma* as a novel biological trait to be considered. However, an absolute concordance between molecular classification and biological characterization has not been detected. The two *T. vaginalis* isolates harboring *Mycoplasma* sp. (S351 and S760) were both classified as group A after MS and SCG determinations; nevertheless, the first isolate was TVV- and the other harbored TVV.

These findings reinforce the two-genotype classification model and suggest potential implications for clinical diagnostics and treatment strategies. The isolates of group A were mostly TVV+ (83.3% rate), sensitive to metronidazole and tinidazole, and could be equated with the Type 1 genotype [7,18,30]. On the other hand, 75% of the isolates clustered in group B were TVV-, including IR78 resistant to 5-nitroimidazoles. Only S351 (TVV-) was included in the first cluster. In this sense, a similar situation can be detected with isolate S019, molecularly associated to group B but TVV+. As indicated above, these discrepancies are consistent with other research studies where no absolute correlation between molecular clustering and biological characteristics was obtained [7,18,29].

Therefore, the above-mentioned results concur with Type 1 and 2 classification: most group A isolates were TVV+ (83.3% rate) and all were metronidazole/tinidazole-sensitive, while 75% of isolates clustered in group B were TVV-, including the 5-nitroimidazole-resistant IR78. It is tempting to speculate that the high number of *T. vaginalis* isolates harboring TVV might occur as the result of a few ancient descendant lineages due to lateral transfer [19]. But also, as data suggest, if harboring these endobionts involves the acquisition of certain advantages in terms of growth, pathogenicity, or even immune survival [11,12,33], it is reasonably a predisposition of *T. vaginalis* to carry these organisms.

It is undoubtedly crucial in clinical practice to know whether the isolate is resistant, and this is especially important for this parasite, for which no effective alternative treatments exist beyond 5-nitroimidazoles. However, it is equally important to emphasize the clinical relevance of determining whether the isolate harbors TVV and/or mycoplasmas, particularly in pregnant women. Recent studies have shown that the treatment of *T. vaginalis* leads to the release of its endobionts, which triggers an inflammatory response upon its recognition by the immune system of the host [11,33]. In the case of pregnant women, the presence of *M. hominis* is directly associated with complications during pregnancy. Furthermore, the release of TVV and the resulting inflammatory response could also pose additional risks (e.g., complicate outcomes in MTZ-treated women or could increase the risk of acquiring HIV which is facilitated by vaginal inflammation) [11,34]. This highlights the need for molecular tools that will enable clinicians to accurately assess the characteristics of the isolate in specific cases, thereby facilitating more tailored and effective treatment strategies.

In this scenario, the identification of a robust, reliable, and affordable molecular method for the classification of *T. vaginalis* in relation to phenotypic features has become one of the main challenges in this field. The identification of an adequate standard for fingerprinting would be of doubtless value as an epidemiological and clinical tool for this neglected STI.

Nevertheless, the main limitations of this study are the number of isolates evaluated, the overall identification of TVV without determining which one(s) of the 5 types described to date it is, and the absence of a robust and reliable marker. Additionally, the detection of TVV by immunofluorescence assay is less specific compared to molecular methods that target viral nucleic acids, which may limit the accuracy of viral identification. It will be of interest in the future to perform a high-throughput screening with many clinical isolates from different geographic origins and to establish statistical associations. In addition, specific identification of the TVV type by PCR and the presence of Ca. M. girerdii would be of value for exploring possible associations. Therefore, we can only take these data collectively and suggest the determination of these endobionts (TVV and *Mycoplasma* sp.) in the biomolecular studies focuses on the identification of an adequate marker for the classification of this parasite.

## 5. Conclusions

To our knowledge, this is the first report that evaluates the possible association between the presence of *Mycoplasma* harbored by *T. vaginalis* and a specific genotypic group based on the combination of a reduced panel of SNPs and MSs with Spanish isolates. The present study corroborates the distribution of the population into two clearly defined groups, based on the reduction to five molecular markers from the panel of twenty-one proposed by other researchers.

Altogether, these preliminary observations may improve the understanding of *T. vaginalis* behavior and could help in the selection of the best reliable and robust method for the classification of isolates based on their biomolecular properties.

## Figures and Tables

**Table 1:** Thermocycler programs used for the amplification of MS markers.

Microsatellite Marker	Initial Denaturation	Amplification Cycle (×30)			Final Extension
		Denaturation	Annealing	Extension	
MS006	95 °C, 3 min	95 °C, 45 s	60 °C, 1 min	72 °C, 2 min	72 °C, 7 min
MS129/MS184	95 °C, 2 min	95 °C, 30 s	47 °C, 10 s	60 °C, 10 s + 65 °C, 45 s	72 °C, 1 min

**Table 2:** Primers used for GP63a and PMS1 amplifications.

Primer	Forward/Reverse	Sequence
GP63D	Forward	5′-TCTAAGATCTCAACAGCCAGAAA
GP63R	Reverse	5′-AATGTCCTTGCCATCTGCTGCAA
PMS1D	Forward	5′-GTCAAAAAAAATTTCAATCAAAATG
PMS1R	Reverse	5′-CTTCCGTCGGACAATTCC

**Table 3:** Phenotypic and molecular results exhibited by the *T. vaginalis* isolates studied.

Characteristics	11	1232	1807	IR78	JH31A4	S/H	S019	S351	S760	S852
MS06	395	395	395	412	395	395	395	395	395	407
MS129	192	192	192	192	192	192	185	192	192	186
MS184	250	241	250	250	250	250	253	250	250	250
MTZ (µg/mL) ^1^	4	2	4	512	4	2	8	8	8	4
TDZ (µg/mL) ^1^	4	4	4	64	4	4	4	4	4	4
TVV	+	-	+	-	+	+	+	-	+	-
*Mycoplasma* sp.	-	-	-	-	-	-	-	+	+	-
Genetic MS Type	MS-A	MS-B	MS-A	MS-B	MS-A	MS-A	MS-B	MS-A	MS-A	MS-B
Genetic GP63a Type	GP63a-A	GP63a-B	GP63a-A	GP63a-B	GP63a-A	GP63a-A	GP63a-B	GP63a-A	GP63a-A	GP63a-B
Genetic PMS1 Type	PMS1-A	PMS1-B	PMS1-A	PMS1-B	PMS1-A	PMS1-A	PMS1-A	PMS1-A	PMS1-A	PMS1-B

^1^ Metronidazole (MTZ) and tinidazole (TDZ) susceptibility is expressed as minimal lethal concentration (MLC).

**Table 4:** Phenotypic and molecular comparison between the *T. vaginalis* isolates characterized by Conrad et al. (2011,2012) [7,9] as Type 1 and 2 and classified according to our criteria (group A and B).

Isolate	Type	MS	GP63a	PMS1	MLC_MTZ_ ^1^	TVV ^1^	*Mycoplasma* ^1^
T1	1	nd	B	B	nd	+	nd
C-1:NIH	1	nd	A	A	8	-	nd
6	1	nd	A	A	nd	+	nd
B7268	1	nd	A	A	nd	+	nd
F1623	2	nd	A	B	nd	-	nd
B7RC2	2	nd	B	B	2	-	nd

^1^ Metronidazole sensitivity exhibited as MLC_MTZ_ and endobiont presence is based on the studies conducted by Conrad et al. (2011, 2012) [7,9]. nd: Not determined.

## Data Availability

The data presented in this study are available from the corresponding author upon request.

## Supplementary Materials

The following supporting information can be downloaded at https://www.mdpi.com/article/10.3390/biology14060618/s1, File S1: Alignment of the GP63 sequences obtained in this study and of the six isolates that have been characterized for both SGP63 and PMS1 markers by Conrad et al. (2011, 2012) [7,9].; File S2: Alignment of PMS1 sequences obtained in this study and of the six isolates that have been characterized for both GP63a and PMS1 markers by Conrad et al. (2011, 2012) [7,9].
