# Supplementary material for: Biomolecular Characterization of Trichomonas vaginalis from Spain: Evaluating Genetic Correlation with Drug Resistance and Endobionts
Source: Biology (Basel). 2025 May 27;14(6):618. doi: 10.3390/biology14060618 (PMC12189323; doi:10.3390/biology14060618)
Supplement: Supplementary file 1 [file biology-14-00618-s001.zip › biology-3557853-supplementary.pdf]

SUPPLEMENTARY FILE S1. Alignment of GP63a sequences obtained in this study and of the six isolates that have been characterized for both GP63a and PMS1 markers by Conrad et al. (2011, 2012) [7,9].

```
                         10        20        30        40        50        60        70        80        90       100
                ---------+---------+---------+---------+---------+---------+---------+---------+---------+---------+
HM365126_T1     AGAACAGGCTTAAATCAAATGCTAAGAATTCATTTTAACACAGACCAATTCAAAGGAGATCAAAAGGAAAACCTTGATAAGATGTTCGATGATATCAAAA   100
HM365125_F1623  .....T.............................................G......................................A.........   100
HM365124_6      .....T.............................................G......................................A.........   100
HM365123_C1NIH  .....T.............................................G......................................A.........   100
HM365122_B7RC2  .....T..............................................................................................   100
HM365121_B7268  .....T.............................................G......................................A.........   100
S760_GP63       .....T.............................................G......................................A.........   100
1232_GP63       .....T.............................................G..C.............................................   100
11_GP63         .....T.............................................G......................................A.........   100
1807_GP63       .....T.............................................G......................................A.........   100
SH_GP63         .....T.............................................G......................................A.........   100
JH31A4_GP63     .....T.............................................G......................................A.........   100
IR78_GP63       .....T..............................................................................................   100
S019_GP63       .....T.............................................G..C.............................................   100
S351_GP63       .....T.............................................G......................................A.........   100
S852_GP63       .....T.............................................G..C.............................................   100


                        110       120       130       140       150       160       170       180       190       200
                ---------+---------+---------+---------+---------+---------+---------+---------+---------+---------+
HM365126_T1     GCTTTTTGAGCTTTTTGAATATTGATACTTCAGAAGTTCAGAATTCTACTTCATTAGCCAGCCTTAAGTCGCATTCTGTCTGCAGTGGTAAATGTAATAG   200
HM365125_F1623  ...............................................G.............................................A......   200
HM365124_6      .......................................................................................A............   200
HM365123_C1NIH  .......................................................................................A............   200
HM365122_B7RC2  .............................................................................................A......   200
HM365121_B7268  ...............................................G.............................................A......   200
S760_GP63       ...............................................G.............................................A......   200
1232_GP63       ...................................................................................G................   200
11_GP63         ...............................................G.............................................A......   200
1807_GP63       ...............................................G.............................................A......   200
SH_GP63         ...............................................G.............................................A......   200
JH31A4_GP63     ...............................................G.............................................A......   200
IR78_GP63       .............................................................................................A......   200
S019_GP63       ....................................................................................................   200
S351_GP63       ...............................................G.............................................A......   200
S852_GP63       ...................................................................................G................   200
```

```
                           210       220       230       240       250       260       270       280       290       300
                  ---------+---------+---------+---------+---------+---------+---------+---------+---------+---------+
HM365126_T1       TGTTCCAAGTAATTTACCAGAAGATGTTGATTTGTACATTCTCGTGAAGCCAGAACCACTAAAAGGTTCTACCCTAGCGCAAGCAGCAGCTATTGATGAT  300
HM365125_F1623    ...............................................................C....................................  300
HM365124_6        ....................................................................................................  300
HM365123_C1NIH    ....................................................................................................  300
HM365122_B7RC2    .......................................................G......C.....................................  300
HM365121_B7268    ...............................................................C....................................  300
S760_GP63         ....................................................................................................  300
1232_GP63         ...................................................G.C.G.......C....................................  300
11_GP63           ..G.................................................................................................  300
1807_GP63         ....................................................................................................  300
SH_GP63           ....................................................................................................  300
JH31A4_GP63       ....................................................................................................  300
IR78_GP63         .......................................................G......C.....................................  300
S019_GP63         ....................................................................................................  300
S351_GP63         ....................................................................................................  300
S852_GP63         .....................................................C.G.......C....................................  300

                           310       320       330       340       350       360       370       380       390       400
                  ---------+---------+---------+---------+---------+---------+---------+---------+---------+---------+
HM365126_T1       TGTCGTACTCAAAGTAAAAGACCTTACGCTGGCTACATCAAAATTAATAGTAACAGAAACATCACAGTCCAGTATCTCAATAGTTCGCACAGAGATTTAA  400
HM365125_F1623    ....................................................................................................  400
HM365124_6        ....................................................................................................  400
HM365123_C1NIH    ....................................................................................................  400
HM365122_B7RC2    ....................................................................................................  400
HM365121_B7268    ....................................................................................................  400
S760_GP63         ....................................................................................................  400
1232_GP63         ....................................................................................................  400
11_GP63           ....................................................................................................  400
1807_GP63         ....................................................................................................  400
SH_GP63           ....................................................................................................  400
JH31A4_GP63       ....................................................................................................  400
IR78_GP63         ....................................................................................................  400
S019_GP63         ....................................................................................................  400
S351_GP63         ....................................................................................................  400
S852_GP63         ....................................................................................................  400
```

```
                          410       420       430       440       450       460       470       480       490       500
                 ---------+---------+---------+---------+---------+---------+---------+---------+---------+---------+
HM365126_T1      TTACGACAATTCTTCATGAATTACAGCATGTCCTTTCATTCTCATCATCCAATTTCGAAAAATGGATCGGATATGACCAAGATAAGGTCAGAAAATCTAT   500
HM365125_F1623   ....................................................................................................   500
HM365124_6       ....................................................................................................   500
HM365123_C1NIH   ....................................................................................................   500
HM365122_B7RC2   ....................................................................................................   500
HM365121_B7268   ....................................................................................................   500
S760_GP63        ....................................................................................................   500
1232_GP63        ....................................................................................................   500
11_GP63          ....................................................................................................   500
1807_GP63        ....................................................................................................   500
SH_GP63          ....................................................................................................   500
JH31A4_GP63      ....................................................................................................   500
IR78_GP63        ....................................................................................................   500
S019_GP63        ....................................................................................................   500
S351_GP63        ....................................................................................................   500
S852_GP63        ....................................................................................................   500

                          510       520       530       540       550       560
                 ---------+---------+---------+---------+---------+---------+------
HM365126_T1      TGTAAATGAAAAATATAATATCACTCAGACATTTCTCATTACGCCACGTCTTAAGGAATGGGTTCA  566
HM365125_F1623   ..................................................................  566
HM365124_6       ..................................................................  566
HM365123_C1NIH   ..................................................................  566
HM365122_B7RC2   ..................................................................  566
HM365121_B7268   ..................................................................  566
S760_GP63        ..................................................................  566
1232_GP63        ..................................................................  566
11_GP63          ..................................................................  566
1807_GP63        ..................................................................  566
SH_GP63          ..................................................................  566
JH31A4_GP63      ..................................................................  566
IR78_GP63        ..................................................................  566
S019_GP63        ..................................................................  566
S351_GP63        ..................................................................  566
S852_GP63        ..................................................................  566
```

SUPPLEMENTARY FILE S2. Alignment of PMS1 sequences obtained in this study and of the six isolates that have been characterized for both GP63a and PMS1 markers by Conrad et al. (2011, 2012) [7,9].

```
                         10        20        30        40        50        60        70        80        90       100
                ---------+---------+---------+---------+---------+---------+---------+---------+---------+---------+
HM365178_T1     TCCCCAGCGCATGCACTCCGAGAGATGATTGAGAACGTATTTGATGCTGGCTGCACACTTTTGACAATTCGAGTTGGAAATGGTGGCCTTGATCATATTT   100
JN380585_T1     ....................................................................................................   100
DQ321767_C1NIH  ...................................................................C................................   100
JN380599_C1NIH  ....................................................................................................   100
HM365177_F1623  ....................................................................................................   100
JN380593_F1623  ....................................................................................................   100
HM365176_6      ....................................................................................................   100
HM365175_B7RC2  ....................................................................................................   100
JN380562_B7RC2  ....................................................................................................   100
HM365174_B7268  ....................................................................................................   100
JN380596_B7268  ....................................................................................................   100
S760_PMS1       ....................................................................................................   100
1232_PMS1       ....................................................................................................   100
11_PMS1         ....................................................................................................   100
1807_PMS1       ....................................................................................................   100
SH_PMS1         ....................................................................................................   100
JH31A4_PMS1     ....................................................................................................   100
IR78_PMS1       ....................................................................................................   100
S019_PMS1       ....................................................................................................   100
S351_PMS1       ....................................................................................................   100
S852_PMS1       ....................................................................................................   100

                        110       120       130       140       150       160       170       180       190       200
                ---------+---------+---------+---------+---------+---------+---------+---------+---------+---------+
HM365178_T1     CAGTATCAGATAATGGACCCGGTATCAGTGAAGAAGGTCTTTCAATGATTTGTGATGAAGGTGTCACATCAAAAGAATTTGGAAAAGATGTAAGTGGCGG   200
JN380585_T1     ....................................................................................................   200
DQ321767_C1NIH  ....................................................................................................   200
JN380599_C1NIH  ....................................................................................................   200
HM365177_F1623  ....................................................................................................   200
JN380593_F1623  ....................................................................................................   200
HM365176_6      ....................................................................................................   200
HM365175_B7RC2  ....................................................................................................   200
JN380562_B7RC2  ....................................................................................................   200
HM365174_B7268  ....................................................................................................   200
JN380596_B7268  ....................................................................................................   200
S760_PMS1       ....................................................................................................   200
1232_PMS1       ....................................................................................................   200
11_PMS1         ....................................................................................................   200
1807_PMS1       ....................................................................................................   200
SH_PMS1         ....................................................................................................   200
JH31A4_PMS1     ....................................................................................................   200
IR78_PMS1       ....................................................................................................   200
S019_PMS1       ....................................................................................................   200
S351_PMS1       ....................................................................................................   200
S852_PMS1       ....................................................................................................   200
```

```
                      210       220       230       240       250       260       270       280       290       300
             ---------+---------+---------+---------+---------+---------+---------+---------+---------+---------+
HM365178_T1  AAGAGGACGAGCATTAGAAGCAATTTCTTATTTATCATATTTAACTATCGACACTTGTACAGATAACAGTAACAATGGTTGGAGATTGCAATTTGATGAA  300
JN380585_T1  ....................................................................................................  300
DQ321767_C1NIH ....................................................................................................  300
JN380599_C1NIH ....................................................................................................  300
HM365177_F1623 ....................................................................................................  300
JN380593_F1623 ....................................................................................................  300
HM365176_6   ....................................................................................................  300
HM365175_B7RC2 ....................................................................................................  300
JN380562_B7RC2 ....................................................................................................  300
HM365174_B7268 ....................................................................................................  300
JN380596_B7268 ....................................................................................................  300
S760_PMS1    ....................................................................................................  300
1232_PMS1    ....................................................................................................  300
11_PMS1      ....................................................................................................  300
1807_PMS1    ....................................................................................................  300
SH_PMS1      ....................................................................................................  300
JH31A4_PMS1  ....................................................................................................  300
IR78_PMS1    ....................................................................................................  300
S019_PMS1    ....................................................................................................  300
S351_PMS1    ....................................................................................................  300
S852_PMS1    ....................................................................................................  300

                      310       320       330       340       350       360       370       380       390       400
             ---------+---------+---------+---------+---------+---------+---------+---------+---------+---------+
HM365178_T1  AATCATACACGAAAAATGGAAAAAATTACGAGACCTAAAGGAACAACAGTTACGGCCCAAACACTATTTTATGGGCAACCAGTTCGTAGACATTATTTTC  400
JN380585_T1  ....................................................................................................  400
DQ321767_C1NIH ..........................................................................................G.........  400
JN380599_C1NIH ....................................................................................................  400
HM365177_F1623 ....................................................................................................  400
JN380593_F1623 ....................................................................................................  400
HM365176_6   ....................................................................................................  400
HM365175_B7RC2 ....................................................................................................  400
JN380562_B7RC2 ....................................................................................................  400
HM365174_B7268 ....................................................................................................  400
JN380596_B7268 ....................................................................................................  400
S760_PMS1    ....................................................................................................  400
1232_PMS1    ....................................................................................................  400
11_PMS1      ....................................................................................................  400
1807_PMS1    ....................................................................................................  400
SH_PMS1      ....................................................................................................  400
JH31A4_PMS1  ....................................................................................................  400
IR78_PMS1    ....................................................................................................  400
S019_PMS1    ....................................................................................................  400
S351_PMS1    ....................................................................................................  400
S852_PMS1    ....................................................................................................  400
```

```
                       410       420       430       440       450       460       470       480       490       500
              ---------+---------+---------+---------+---------+---------+---------+---------+---------+---------+
HM365178_T1   TTGAACATAAATCGCAGCAAGTTTCAGATATAATCGAAATTACACAATCATTTGCAATTGCAAGCTCAGCCAATATGACCGTTACTCTCGATAACAAATT  500
JN380585_T1   ....................................................................................................  500
DQ321767_C1NIH ....................................................................................................  500
JN380599_C1NIH ....................................................................................................  500
HM365177_F1623 ....................................................................................................  500
JN380593_F1623 ....................................................................................................  500
HM365176_6    ....................................................................................................  500
HM365175_B7RC2 ....................................................................................................  500
JN380562_B7RC2 ....................................................................................................  500
HM365174_B7268 ....................................................................................................  500
JN380596_B7268 ....................................................................................................  500
S760_PMS1     ....................................................................................................  500
1232_PMS1     ....................................................................................................  500
11_PMS1       ....................................................................................................  500
1807_PMS1     ....................................................................................................  500
SH_PMS1       ....................................................................C...............................  500
JH31A4_PMS1   ....................................................................................................  500
IR78_PMS1     ....................................................................................................  500
S019_PMS1     ....................................................................................................  500
S351_PMS1     ....................................................................................................  500
S852_PMS1     ....................................................................................................  500

                       510       520       530       540       550       560       570       580       590       600
              ---------+---------+---------+---------+---------+---------+---------+---------+---------+---------+
HM365178_T1   ACTCATACAAGTATCCACTCCAAAACGTGATAGCAGGATCAGTACCGTTTTTGGATCAGATATCATGAAAGGATTTGAACATGGAAAAGTTTCTCTTGAT  600
JN380585_T1   ....................................................................................................  600
DQ321767_C1NIH .....................................................................................G..............  600
JN380599_C1NIH .....................................................................................G..............  600
HM365177_F1623 .....................................................................................G..............  600
JN380593_F1623 .....................................................................................G..............  600
HM365176_6    .....................................................................................G..............  600
HM365175_B7RC2 .....................................................................................G..............  600
JN380562_B7RC2 .....................................................................................G..............  600
HM365174_B7268 .....................................................................................G..............  600
JN380596_B7268 .....................................................................................G..............  600
S760_PMS1     .....................................................................................G..............  600
1232_PMS1     ....................................................................................................  600
11_PMS1       .....................................................................................G..............  600
1807_PMS1     .....................................................................................G..............  600
SH_PMS1       .....................................................................................G..............  600
JH31A4_PMS1   .....................................................................................G..............  600
IR78_PMS1     .....................................................................................G..............  600
S019_PMS1     .....................................................................................G..............  600
S351_PMS1     .....................................................................................G..............  600
S852_PMS1     ....................................................................................................  600
```

```
                          610       620       630       640       650       660       670       680       690       700
                ---------+---------+---------+---------+---------+---------+---------+---------+---------+---------+
HM365178_T1     AAATGGCATTCAGAAGCTTCTCTTGAATATTACACAGCTACTCCTACAGCAAATTCCAACAAAAAAATCTTATTTATTGTTAATAATCGACCATGTGTTT 700
JN380585_T1     .................................................................................................... 700
DQ321767_C1NIH  .........C.....................................G.................................................... 700
JN380599_C1NIH  .........C.......................................................................................... 700
HM365177_F1623  ...................................................C................................................ 700
JN380593_F1623  ...................................................C................................................ 700
HM365176_6      .................................................................................................... 700
HM365175_B7RC2  ...................................................C................................................ 700
JN380562_B7RC2  ...................................................C................................................ 700
HM365174_B7268  .........C.......................................................................................... 700
JN380596_B7268  .........C.......................................................................................... 700
S760_PMS1       .........C.......................................................................................... 700
1232_PMS1       .................................................................................................... 700
11_PMS1         .........C.......................................................................................... 700
1807_PMS1       .........C.......................................................................................... 700
SH_PMS1         .........C.......................................................................................... 700
JH31A4_PMS1     .........C.......................................................................................... 700
IR78_PMS1       .................................................................................................... 700
S019_PMS1       .........C.......................................................................................... 700
S351_PMS1       .........C.......................................................................................... 700
S852_PMS1       .................................................................................................... 700

                          710       720       730       740       750       760       770       780       790       800
                ---------+---------+---------+---------+---------+---------+---------+---------+---------+---------+
HM365178_T1     GTTCTGGACTTTATCGCGCAATCAAAAACGAATTTAGACTTTGTGCAGGACCGAAACTACCATCAGGAGTATTCTTTATTACTGCTCCACGAAATACATT 800
JN380585_T1     .................................................................................................... 800
DQ321767_C1NIH  .................................................................................................... 800
JN380599_C1NIH  .................................................................................................... 800
HM365177_F1623  .................................................................................................... 800
JN380593_F1623  .................................................................................................... 800
HM365176_6      .................................................................................................... 800
HM365175_B7RC2  .................................................................................................... 800
JN380562_B7RC2  .................................................................................................... 800
HM365174_B7268  .................................................................................................... 800
JN380596_B7268  .................................................................................................... 800
S760_PMS1       .................................................................................................... 800
1232_PMS1       .................................................................................................... 800
11_PMS1         .................................................................................................... 800
1807_PMS1       .................................................................................................... 800
SH_PMS1         .................................................................................................... 800
JH31A4_PMS1     .................................................................................................... 800
IR78_PMS1       .................................................................................................... 800
S019_PMS1       .................................................................................................... 800
S351_PMS1       .................................................................................................... 800
S852_PMS1       .................................................................................................... 800
```

```
                         810       820       830       840       850       860       870       880       890       900
                ---------+---------+---------+---------+---------+---------+---------+---------+---------+---------+
HM365178_T1     TGATTTTATACCGAATTCACCTCTTATTTCGATTTCTTTTGAGCACGAAAATATTCTACAGAAGACATTTTGTGAAATATTAAGCTCCGTTTGGAAGAAA   900
JN380585_T1     ....................................................................................................   900
DQ321767_C1NIH  ....................................................................................................   900
JN380599_C1NIH  ....................................................................................................   900
HM365177_F1623  .......................................................................................A............   900
JN380593_F1623  .......................................................................................A............   900
HM365176_6      ....................................................................................................   900
HM365175_B7RC2  .......................................................................................A............   900
JN380562_B7RC2  .......................................................................................A............   900
HM365174_B7268  ....................................................................................................   900
JN380596_B7268  ....................................................................................................   900
S760_PMS1       ....................................................................................................   900
1232_PMS1       ....................................................................................................   900
11_PMS1         ....................................................................................................   900
1807_PMS1       ....................................................................................................   900
SH_PMS1         ....................................................................................................   900
JH31A4_PMS1     ....................................................................................................   900
IR78_PMS1       .......................................................................................A............   900
S019_PMS1       ....................................................................................................   900
S351_PMS1       ....................................................................................................   900
S852_PMS1       ....................................................................................................   900

                         910       920       930       940       950       960       970       980       990      1000
                ---------+---------+---------+---------+---------+---------+---------+---------+---------+---------+
HM365178_T1     TCCAGTGAAAAATTGACATTTAACAATGTAATCTCAGAAGAACCGATTAGATCACTTCCACAGATGACCAGACTGAAGAACACCGAGCCATTAGGTATAG   1000
JN380585_T1     ....................................................................................................   1000
DQ321767_C1NIH  ....................................................................................................   1000
JN380599_C1NIH  ....................................................................................................   1000
HM365177_F1623  ....................................................................................................   1000
JN380593_F1623  ....................................................................................................   1000
HM365176_6      ....................................................................................................   1000
HM365175_B7RC2  ....................................................................................................   1000
JN380562_B7RC2  ....................................................................................................   1000
HM365174_B7268  ....................................................................................................   1000
JN380596_B7268  ....................................................................................................   1000
S760_PMS1       ....................................................................................................   1000
1232_PMS1       ....................................................................................................   1000
11_PMS1         ....................................................................................................   1000
1807_PMS1       ....................................................................................................   1000
SH_PMS1         ....................................................................................................   1000
JH31A4_PMS1     ....................................................................................................   1000
IR78_PMS1       ....................................................................................................   1000
S019_PMS1       ....................................................................................................   1000
S351_PMS1       ....................................................................................................   1000
S852_PMS1       ....................................................................................................   1000
```

```
                        1010      1020      1030      1040      1050      1060      1070      1080      1090
               ---------+---------+---------+---------+---------+---------+---------+---------+---------+--------
HM365178_T1    AATGTCATTGCCAAGCTACAACAGATGATATTTTGGAAAGATTTAAAAATTGCCAAAATTATGTACCAGATTATGGACTATCCTACGATGCTATTGAG
1098
JN380585_T1    ..................................................................................................   1098
DQ321767_C1NIH ..................................................................................................   1098
JN380599_C1NIH ..................................................................................................   1098
HM365177_F1623 ..................................................................................................   1098
JN380593_F1623 ..................................................................................................   1098
HM365176_6     ..................................................................................................   1098
HM365175_B7RC2 ..................................................................................................   1098
JN380562_B7RC2 ..................................................................................................   1098
HM365174_B7268 ..................................................................................................   1098
JN380596_B7268 ..................................................................................................   1098
S760_PMS1      ..................................................................................................   1098
1232_PMS1      ..................................................................................................   1098
11_PMS1        ..................................................................................................   1098
1807_PMS1      ..................................................................................................   1098
SH_PMS1        ..................................................................................................   1098
JH31A4_PMS1    ..................................................................................................   1098
IR78_PMS1      ..................................................................................................   1098
S019_PMS1      ...........................................A....T................................................   1098
S351_PMS1      ..................................................................................................   1098
S852_PMS1      ..................................................................................................   1098
```
